# Integrating Circadian Activity and Gene Expression Profiles to Predict Chronotoxicity of *Drosophila suzukii* Response to Insecticides

**DOI:** 10.1371/journal.pone.0068472

**Published:** 2013-07-05

**Authors:** Kelly A. Hamby, Rosanna S. Kwok, Frank G. Zalom, Joanna C. Chiu

**Affiliations:** Department of Entomology and Nematology, College of Agricultural and Environmental Sciences, University of California Davis, Davis, California, United States of America; United States Department of Agriculture, Agriculture Research Service, United States of America

## Abstract

Native to Southeast Asia, *Drosophila suzukii* (Matsumura) is a recent invader that infests intact ripe and ripening fruit, leading to significant crop losses in the U.S., Canada, and Europe. Since current *D. suzukii* management strategies rely heavily on insecticide usage and insecticide detoxification gene expression is under circadian regulation in the closely related *Drosophila melanogaster*, we set out to determine if integrative analysis of daily activity patterns and detoxification gene expression can predict chronotoxicity of *D. suzukii* to insecticides. Locomotor assays were performed under conditions that approximate a typical summer or winter day in Watsonville, California, where *D. suzukii* was first detected in North America. As expected, daily activity patterns of *D. suzukii* appeared quite different between ‘summer’ and ‘winter’ conditions due to differences in photoperiod and temperature. In the ‘summer’, *D. suzukii* assumed a more bimodal activity pattern, with maximum activity occurring at dawn and dusk. In the ‘winter’, activity was unimodal and restricted to the warmest part of the circadian cycle. Expression analysis of six detoxification genes and acute contact bioassays were performed at multiple circadian times, but only in conditions approximating Watsonville summer, the cropping season, when most insecticide applications occur. Five of the genes tested exhibited rhythmic expression, with the majority showing peak expression at dawn (ZT0, 6am). We observed significant differences in the chronotoxicity of *D. suzukii* towards malathion, with highest susceptibility at ZT0 (6am), corresponding to peak expression of cytochrome P450s that may be involved in bioactivation of malathion. High activity levels were not found to correlate with high insecticide susceptibility as initially hypothesized. Chronobiology and chronotoxicity of *D. suzukii* provide valuable insights for monitoring and control efforts, because insect activity as well as insecticide timing and efficacy are crucial considerations for pest management. However, field research is necessary for extrapolation to agricultural settings.

## Introduction

The recently introduced and rapidly spreading Spotted Wing Drosophila (*Drosophila suzukii* Matsumura) has unique anatomy among *Drosophila* species that enables it to become a serious economic pest [1]–[4]. Female *D. suzukii* have a serrated ovipositor and exhibit a preference for ovipositing in ripe and ripening intact fruit as opposed to the overripe and blemished fruit that other *Drosophila* species are known to infest [3], [5]. Since its initial detection in the continental United States (U.S.) in 2008 in the berry-growing central coastal region of California, significant crop losses have been reported not only in California, but also throughout the U.S., Canada, and Europe among growers of berry crops (e.g. caneberry) and soft-skinned stone fruits (e.g. cherry) [6]–[8]. At 20% damage, an estimated 300 million dollars annually could be lost to Spotted Wing Drosophila in California alone [9]. *Drosophila* flies oviposit directly into the fruit and the larvae live within the fruit; therefore, the adult is the only stage that can be targeted for control by conventional pesticides [5].

The most commonly used insecticides for control of *D. suzukii* (organophosphates, pyrethroids, and spinosyns) have good contact although varying residual field activity that typically lasts longer than three days [10], [11]. Targeting pesticide sprays to peaks of fly activity increases the likelihood of direct contact mortality, especially for products with marginal efficacy. Previous chronotoxicity work has suggested that time of greatest insecticide susceptibility corresponds with the onset of a time of increased activity in the insect [12]. Casual field observations indicate that *D. suzukii* activity is greatest in warm parts of the day, with little activity observed on extremely hot or cold days. This is not surprising, as daily activity is not only impacted by daily light/dark cycles, or photoperiod, but also by temperature [13], [14]. However, systematic studies of *D. suzukii* activity patterns have not been performed to date. On the other hand, rigorous measurement of daily activity patterns of a closely related model organism, *Drosophila melanogaster*, is commonly used as an output parameter for accessing the functionality of the endogenous circadian clock, which drives daily rhythms in animal physiology, metabolism, and behavior [15], [16]. Locomotor assays in *D. melanogaster* are regularly performed at constant temperature with abrupt changes during light/dark transitions [17], [18]. Under these conditions, *D. melanogaster* typically has a bimodal activity distribution that is described as “crepuscular” as the flies anticipate lights on, i.e. morning activity peak, and lights off, i.e. evening activity peak, but appear passive during the mid-day ‘siesta’. However, with more natural temperature gradient cycles and gradual changes in light intensity, it has been found both in laboratory-simulated and outdoor conditions that *D. melanogaster* may also have an afternoon peak of activity in place of the ‘siesta’ period, at least in some environmental regimes [19]. In addition, the circadian clocks of insects also impact seasonal changes in activity patterns, as well as other processes such as migration and diapauses [13], [20], [21]. Seasonal changes in photoperiod as well as daily temperature cycles have been shown to modulate the timing and amplitudes of activity peaks [22]–[27]. Little is known about the seasonal biology of *D. suzukii* in its native range, making it difficult to predict their biology in berry-growing regions over the growing season. Translations of Japanese literature suggest that *D. suzukii* overwinter as adults and disappear during the coldest parts of the winter [28]. It is unclear whether they are in diapause or possibly capable of reproducing during this time if conditions become more favorable, i.e. milder temperatures and more abundant food sources. Since *D. suzukii* occupy a very different habitat than the majority of Drosophilidae, with its preference for fresh fruit, it is possible that they have evolved somewhat different biology and activity patterns than those of *D. melanogaster*.

Daily activity rhythm is only one of the many biological processes that are controlled by the circadian clock. The clock manifests rhythms in physiology and behavior by regulating cyclic expression of genes with periods of 24 hours. Analysis of *D. melanogaster* transcriptomes using DNA microarrays and more recently RNA sequencing, has identified a large number of clock-controlled cycling genes that are involved in diverse physiological processes [29]–[37]. Genes encoding detoxification enzymes of xenobiotics and endogenous compounds are well represented in all of these studies, suggesting that metabolism and detoxification of insecticides is likely to be under circadian clock control. The detoxification process is known to occur in two major phases, followed by a third excretory phase. Phase I detoxification refers to primary metabolism and alters basic chemical structure [38]. Phase I redox reactions catalyzed by cytochrome P450-dependent monooxygenases (CYPs) are considered to be the only metabolic system in insects that can mediate resistance to all classes of insecticides [39], [40]. These enzymes are a broad group of isozymes that vary in protein abundance and substrate specificity, but utilize identical oxidizing systems [38], [41]. Phase II reactions, often called conjugation reactions, usually produce hydrophilic products by modification of existing reactive functional groups creating products that are more easily secreted, and are often catalyzed by UDP-glycosyltransferases (UGTs) and glutathione S-transferases (GSTs) [38]. GSTs are implicated in insect resistance to organophosphate, organochlorine, and pyrethroid insecticides [40], [42]. Esterases are another important group of metabolic enzymes that are frequently implicated in the resistance of insects to organophosphates, carbamates, and pyrethroids [40]. Phase III of detoxification involves the active export of the metabolized toxins via transmembrane transporters [43]. In some cases, excretion of metabolized toxins occur after Phase I reactions as well. Since metabolic resistance of insecticides is believed to develop through upregulation of detoxification pathways [40], [44], the time of day when expression of detoxification genes and subsequent enzyme activities is at their minimum may correspond to the time when insects are most susceptible to insecticides. A study using *D. melanogaster* as a model concluded that there is indeed some correlation between the circadian timing of susceptibility to certain classes of pesticides to expression of circadian regulated Phase I and II detoxification enzymes [45], [46]. Assuming that detoxification of xenobiotics in *D. suzukii* is similar to that in *D. melanogaster*, other insects, and even mammals [46]–[49] in being under circadian clock control, it may be possible to identify a time period when *D. suzukii* is most susceptible to insecticides.

To examine whether integrative analysis of daily activity patterns and circadian gene expression of different classes of detoxification genes can predict chronotoxicity of *D. suzukii* to chemical control, we chose the *D. suzukii* population from Watsonville, California, U.S. as a test case. We performed *Drosophila* locomotor activity assays [17], [18] at conditions that approximate summer (July/August) and winter (January/February) conditions in Watsonville, one of California’s main berry production areas and site of the first *D. suzukii* detection in North America [2], [9]. An organophosphate insecticide (malathion) and a pyrethroid (fenpropathrin) insecticide were chosen for our experiments based on prevalence of use in 2010 California strawberry fields [50] and efficacy for control of *D. suzukii* [10]. Insecticides from these two classes remain some of the most well-known and understood insecticides in terms of mode of action and mechanisms of detoxification [51], [52]. They were applied as contact chronotoxicity bioassays at Watsonville summer conditions, when most pesticide applications take place, and the results were corroborated to expression analysis of detoxification genes, some of which were previously found to be under clock regulation in *D. melanogaster* [31], [45], [46], [49]. Integrating the results of our chronobiology and chronotoxicity experiments will lead to a better understanding of the physiology of *D. suzukii* and may lead to more effective control of this invasive pest.

## Materials and Methods

### Fly Strains and Rearing

The *D. suzukii* used in our studies were originally collected as adults from Watsonville, CA, U.S.A. in September 2009. Permission for fly collection was granted by Garroutte Farms, Inc. (Watsonville, CA). Isofemale lines from this collection were mixed and maintained in Fisherbrand square, polyethylene, 6 oz. stock bottles (Fisher Scientific, Pittsburgh, PA) containing 50 ml of Applied Scientific Jazz-Mix *Drosophila* Food (Fisher Scientific, Pittsburgh, PA) or Bloomington stock center fly food recipe. Colonies were kept between 22°C to 25°C in a cabinet incubation chamber (Percival Scientific, Inc., Perry, IA) with a 12:12 h light:dark cycle.

### Analysis of Locomotor Activity Rhythms

**Figure 1:**
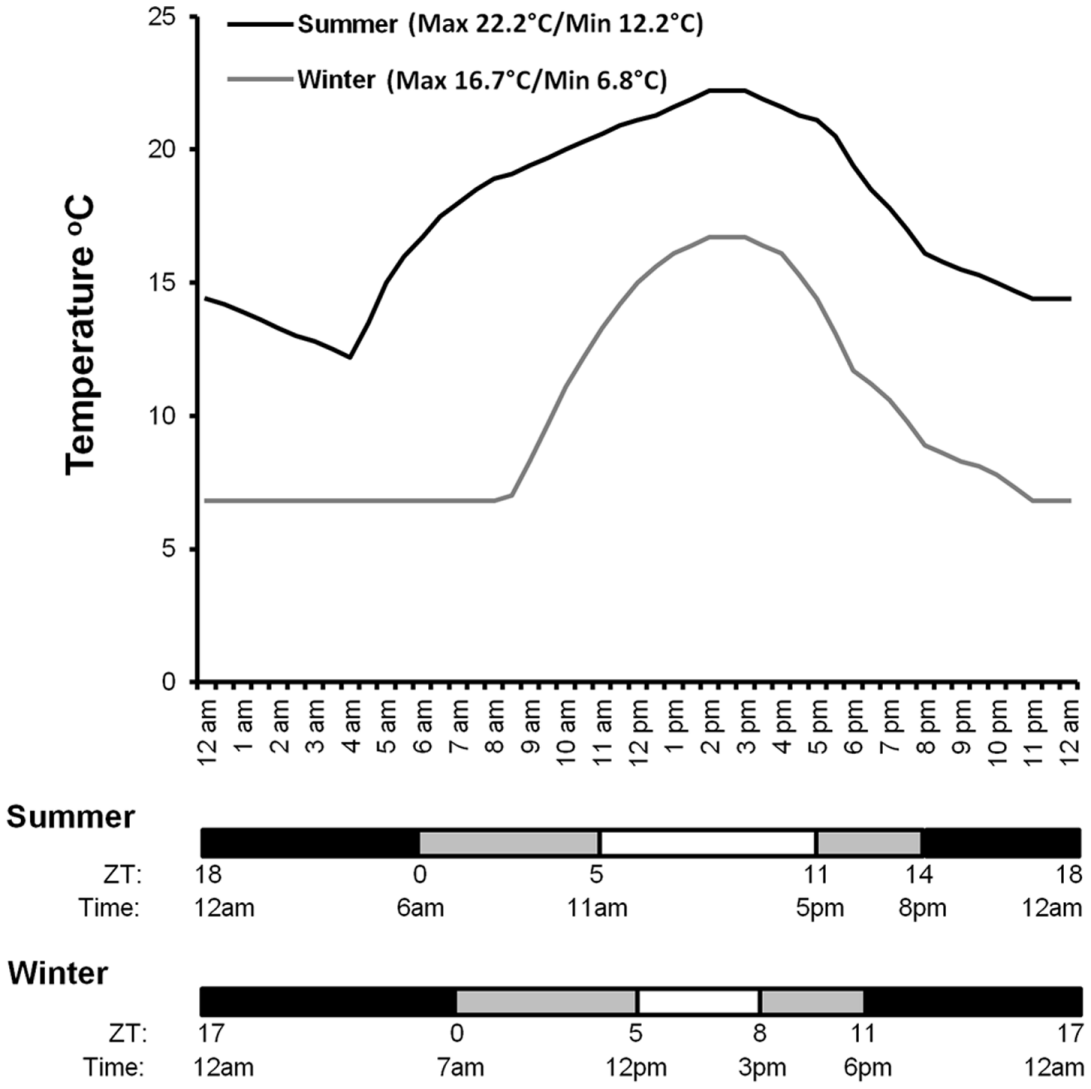
Percival biological incubator programs to approximate Watsonville, CA, U.S.A. environmental conditions. Incubator settings to mimic a typical summer (July/August) or winter (January/Feburary) day in Watsonville, CA. The two environmental settings are indicated in black (summer) and dark gray (winter) respectively. Zeitgeber time and light intensity are shown on the X-axis for both summer and winter days. Light intensity is classified as bright (both banks of lights on in the incubator); dim (only one out of two banks of lights on); and dark (all lights off). These three levels of light intensity are indicated by the horizontal bars beneath the graph. Black bars = lights off; dark gray bars = dim light; white bar = bright light. Both zeitgeber (ZT) and natural time for changes in light intensity are shown underneath the horizontal bars. Summer: Lights-on time (ZT0) is at 6am and lights-off time (ZT14) is at 8pm. Winter: Lights-on time is at 7am (ZT0) and lights-off time (ZT11) is at 6pm. Temperature fluctuations over the circadian day are indicated on the Y-axis. The temperature ranges for summer and winter simulations are 12.2°C to 22.2°C and 6.8°C to 16.7°C respectively. Due to the low survivorship of *D. suzukii* flies at low [57], the lowest temperature for the winter day is set at 6.8°C. On average, winter nights can go down to 4°C. Peak temperature of 22.2°C in the summer occurs between 2pm (ZT8) and 3:30pm (ZT9.5). Peak temperature of 16.7°C in the winter occurs between 2pm (ZT7) and 3:30pm (ZT8.5).

Virgin female *D. suzukii*, selected to prevent the presence of larvae in activity tubes, and male *D. suzukii* were subjected to a locomotor activity assay using the *Drosophila* Activity Monitoring System (DAMS) (Trikinetics, Inc., Waltham, MA) at conditions that were designed to resemble summer (July/August; 14:10 h light:dark cycle; Max 22.2°C/Min 12.2°C) and winter (January/February; 11:13 h light:dark cycle; Max 16.7°C/Min 6.8°C) day-night as well as temperature cycles in Watsonville (Figure 1 and Table S1 in File S1). Although the experiments were conducted in controlled environmental chambers, the temperature cycles were designed to more closely resemble the observed natural field conditions, with the temperatures set to change gradually, e.g. in [19], [24], [26]. The two temperature regimes used in these experiments reached night-time temperatures that were generally much lower than the lowest temperature used in previous laboratory studies that assay activity rhythms in *D. melanogaster*, which is generally around 18°C, e.g. in [23], [25], [26], [53]. The night-time low for the ‘summer’ condition is 12.2°C, while the ‘winter’ night-time low is 6.8°C. Due to technical limitation of the environmental incubators used, gradual changes in light intensity throughout the day were not feasible, but we simulated three different levels of light intensity by controlling the number of fluorescent bulbs that were turned on. Mid-day and early afternoon sun, which was classified as ‘bright’ was simulated by having 2 out of 2 banks of lights on in the incubator; morning and late afternoon sun, classified as ‘dim’ was simulated by having 1 out of 2 banks of lights on; finally, night-time was simulated by having all lights off. Activity rhythms of male and female flies were monitored for at least seven circadian day/night cycles. Fly activity monitoring using DAMS and data analysis using FaasX were as previously described in Chiu et al. [18]. The first full day of the recording period was not included in the analysis to allow for acclimation of the flies to treatment conditions and stabilization of activity patterns.

### Expression Analysis of Detoxification Genes

*Drosphila suzukii* adults (mixed sexes) removed from the colony were entrained in Watsonville ‘summer’ (light/dark cycle and temperature cycle) conditions (Figure 1 and Table S1 in File S1) for three days, then collected at 6 time points: ZT 0 (6am), 4 (10am), 8 (2pm), 12 (6pm), 16 (10pm), and 20 (2am), on the fourth day and frozen immediately on dry ice. Approximately 40 to 50 flies were collected at each time point. Fly bodies (thorax and abdomen regions) were separated from heads and collected using metal sieves. RNA extraction was performed as follows: 3X volume of TRI-reagent (Sigma Aldrich, St Louis, MO) was added to fly bodies and homogenized by grinding using a motorized pestle. One-fifth volume of 100% chloroform was added to each sample and incubated at room temperature for 10 minutes. Samples were spun down at 13,000 rpm for 15 minutes at 4°C. The upper aqueous layer was collected and nucleic acid was precipitated by adding an equal volume of 100% isopropanol and incubated at room temperature for 10 minutes. Samples were spun down at 13,000 rpm for 15 minutes at 4°C, and pellets were washed once with two volumes of 70% ethanol. RNA samples were treated with RQ1 RNase-free DNase (Promega, Madison, WI) according to manufacturer’s protocol. RNA concentrations were quantified using a Biophotometer (Eppendorf, Hauppauge, NY). 4 µg of total RNA was used to synthesize cDNA using ThermoScript RT-PCR System (Life Technologies, Grand Island, NY) according to the manufacturer’s protocol. Dilutions (1:10) of cDNA samples were used in quantitative real-time PCR reactions. Gene-specific primers (see Materials and Methods S1 in File S1) were designed based on sequence analysis using a *D. suzukii* genome scaffold (unpublished; KA Hamby et al.), and optimized at an annealing temperature of 60°C. Melt curve and BLAST analysis were used as criteria to determine primer specificity. The quantitative real-time PCR assays were performed using SsoAdvanced SYBR Green Supermix (Bio-rad, Hercules, CA) in a CFX96 Touch Real-Time PCR Detection thermal cycler (Bio-Rad, Hercules, CA). Cycling conditions were 95°C for 30 seconds, 40 cycles of 95°C for 5 seconds, followed by an annealing/extension phase at 60°C for 30 seconds. The reaction was concluded with a melt curve analysis going from 65°C to 95°C in 0.5°C increments at 5 seconds per step. Three technical replicates were performed for each data point of each biological replicate, and at least three biological replicates were performed for analysis of each gene. Data were analyzed using the standard ΔΔCt method and target gene mRNA expression levels were normalized to the reference gene mRNA levels, which remain unchanged over a circadian day. Finally, Ct values for all time points were divided by the highest Ct value to generate a scale from 0 to 1.

### Chronotoxicity Bioassays

Ten to twelve 2–3 day old adult female *D. suzukii* collected from the previously described colony were placed in clean diet vials with fruit fly media (Standard Bloomington media without optional malt extract, http://flystocks.bio.indiana.edu/Fly_Work/media-recipes/bloomfood.htm) and placed in a biological incubator (Percival Scientific Inc., Perry, IA) programmed to the Watsonville ‘summer’ condition (Figure 1 and Table S1 in File S1) to acclimate for approximately 50 hours. At the appropriate Zeitgeber time (ZT) point, they were introduced to insecticide-coated 20 ml scintillation vials (approximate surface area: 56.9 cm^2^), then placed back into the incubator for 1 hour (±5 min). Vials were coated with 200 µl of pesticide solution by rolling until dry on an Old Fashioned Hot Dog Roller (The Helman Group Ltd., Oxnard, CA) at room temperature 1 hour prior to the ZT time point. Caps were coated with 50 µl of pesticide solution and allowed to dry in a fume hood. Danitol 2.4 EC (fenpropathrin, Valent U.S.A. Corporation, Walnut Creek, CA) and Malathion 8 (malathion, Gowan Company, Yuma, AZ) were provided from their respective manufacturers for use in this trial. All pesticide solutions included acetone as a solvent to improve evaporation and coating. Control vials were coated with acetone alone. For each day of experimentation, each pesticide dose was replicated in three independent vials at each of four Zeitgeber time points: ZT 0 (6am), 6 (12pm), 12 (6pm), and 20 (2am). Total number of flies assayed for all doses and time points are shown in Table 1. Due to the large sample size, only six pesticide doses and a control treatment were performed for each day of experimentation, explaining the higher number of flies assayed for the control treatment. At least 2 independent days of experimentation were performed for all doses, with 3 independent days performed for most doses. A total of 11 doses for malathion and 12 for fenpropathrin were performed (Table 1). Range-finding experiments at ZT 12 were used to choose the doses for each pesticide. For time points that were in the lights-off part of the light/dark cycle, flies were loaded into the pesticide vials and returned to the clean diet vials in a dark room under red light. Mortality was assessed 72 hours post treatment and moribund flies, those that were upside down and hardly moving, were considered dead for the purpose of this bioassay.

**Table 1:** Total female *D. suzukii* dosed for each insecticide (field rate of active ingredient (AI)) at each vial concentration (µmol AI/cm^2^), Zeitgeber time point, and replicate.

Malathion 8 (Malathion 0.0692 µmol AI/cm^2^)														Danitol 2.4 EC (Fenpropathrin 0.0117 µmol AI/cm^2^)																
		ZT0			ZT6			ZT12			ZT20					ZT0			ZT6					ZT12				ZT20		
µmol/cm^2^	% FieldRate	1	2	3	1	2	3	1	2	3	1	2	3	µmol/cm^2^	% Field Rate	1	2	3		1	2	3	1		2	3	1		2	3
0.00E+00	0.00	93	101	99	101	101	89	97	98	97	91	95	98	0.00E+00	0.00	75	72	73		71	72	71	71		69	70	70		69	71
1.18E-05	0.02	22	23	23	23	22	23	23	22	23	23	22	24	4.83E-05	0.41	32	36	34		35	36	36	35		33	35	33		36	36
2.11E-05	0.03	47	46	47	43	45	47	47	45	47	47	40	45	8.39E-05	0.72	36	36	36		34	34	34	33		36	37	31		36	36
3.33E-05	0.05	21	23	23	24	23	23	23	22	24	23	24	23	1.24E-04	1.06	36	35	36		34	35	34	36		24	47	35		35	35
3.75E-05	0.05	21	23	20	22	23	23	24	22	24	20	21	22	1.52E-04	1.30	34	35	36		36	36	35	47		24	35	36		36	35
5.28E-05	0.08	24	22	24	22	23	24	23	21	24	24	24	23	1.56E-04	1.33	35	36	37		34	35	35	34		35	34	35		39	37
8.37E-05	0.12	24	24	20	23	24	24	22	23	23	24	23	23	1.96E-04	1.68	35	36	36		36	35	36	36		36	36	34		36	36
1.18E-04	0.17	19	23	23	22	24	24	24	25	23	22	20	24	2.47E-04	2.11	35	36	36		35	37	34	35		35	36	35		36	35
1.33E-04	0.19	22	24	24	24	23	22	22	23	22	24	24	27	2.77E-04	2.38	35	36	36		34	35	36	36		34	35	36		36	36
2.11E-04	0.30	69	65	64	71	73	70	66	65	69	71	68	65	3.11E-04	2.66	36	35	36		35	36	35	34		35	34	36		36	36
2.83E-04	0.41	30	32	32	29	30	32	32	30	32	26	29	25	3.91E-04	3.35	38	36	35		36	35	36	35		35	36	35		35	36
3.75E-04	0.54	79	72	83	78	80	80	80	81	76	69	77	77	5.05E-04	4.32	36	35	36		36	35	36	36		36	36	36		36	37
														9.19E-04	7.86	35	35	35		35	35	36	35		34	36	36		37	36

The mortality data for each insecticide was pooled by replicate vials (vial 1, 2, or 3 as listed in Table 1) such that the total number of flies assayed at each time point and dose (vial concentration in µmol active ingredient (AI)/cm^2^) was the response across all days of experimentation performed. We anticipate high variability for our data as we used formulated product and a *D. suzukii* population with heterogeneous genetic background to closely approximate field conditions. First, a mixed model logistic regression was performed in SAS 9.2 (SAS Institute 2008) to account for variability and ensure that time point had a significant effect. Once this was verified, fitting of dose-response curves and calculation of LC_50_ was performed using PRISM v.5.0 (GraphPad Software, Inc., La Jolla, CA). A variable slope sigmoidal dose response curve was fit to the data (Table 2), with a four parameter logistic equation where Y = BOTTOM+(TOP-BOTTOM)/(1+10∧((LOGEC50-X)*H)) and EC50 is the midpoint of the curve, H is the Hill slope, Y is response values and X is Log(Dose). Response values were baseline-corrected to the control by defining the control response as 0% and were also normalized by defining 100% response as the highest concentration’s mortality value, therefore the curve fitting analysis was constrained to a minimum of 0 (BOTTOM) and maximum of 100 (TOP). Normality of residuals was tested using the Shapiro-Wilk test. Although ZT20 had a Shapiro-Wilk W-values of 0.90, no further transformation was performed as it was the only time point to deviate. The response curves at each time point were compared using an *F*-test of both curve-fit parameters (slope and LC_50_) simultaneously in PRISM v. 5.0.

**Table 2:** Normalized variable slope sigmoidal dose response curve fit, slope, and LC_50_ values (µmol active ingredient (AI)/cm^2^ **)** for each insecticide at different Zeitgeber time points (N = data pooled across treatment days to each replicate vial).

		Malathion^a^				Fenpropathrin^b^			
Time		N	R^2^	Slope ± SE	LC_50_ (95% CI)	N	R^2^	Slope ± SE	LC_50_ (95% CI)
ZT0	6am	3	0.7305	2.13±0.52	9.25E-5 (7.15E-5–1.20E-4)	3	0.8809	2.48±0.30	1.77E-4(1.61E-4–1.94E-4)
ZT6	12pm	3	0.8173	2.37±0.47	1.01E-4 (8.42E-5–1.22E-4)	3	0.8840	2.32±0.27	1.82E-4 (1.66E-4–2.00E-4)
ZT12	6pm	3	0.8664	2.93±0.57	1.21E-4 (1.06E-4–1.39E-4)	3	0.8905	1.89±0.19	1.85E-4 (1.68E-4–2.03E-4)
ZT20	2am	3	0.8923	4.76±1.13	1.44E-4 (1.29E-4–1.60E-4)	3	0.8295	1.73±0.21	1.55E-4 (1.37E-4–1.76E-4)

a H_0_ 2 parameters (Slope and LC_50_) same for all data sets; *F*_6,124_ = 3.52, *P* = 0.0030.

b H_0_ 2 parameters (Slope and LC_50_) same for all data sets; *F*_6,136_ = 2.04, *P* = 0.0644.

## Results

### Circadian Locomotor Activity Profile of Drosophila Suzukii Changes with Seasonal Variations in Photoperiod and Temperature Cycles

Since there is evidence that insecticide susceptibility corresponds with activity rate of the target insect due to contact toxicity [12], we set out to determine the activity patterns of *D. suzukii* using the *Drosophila* Activity Monitoring System (DAMS). Anticipating that activity patterns will vary over the long California berry-growing season with changes in photoperiod and daily temperature cycles, we subjected male and female *D. suzukii* flies to locomotor activity assays in environmental conditions, i.e. photoperiod and temperature cycle, that approximate either summer (July/August; 14hr:10hr light/dark cycle; Max 22.2°C/Min 12.2°C) or winter (January/February; 11hr:13hr light/dark cycle; Max 16.7°C/Min 6.8°C) of Watsonville, CA, a major berry-growing region (Figure 1 and Table S1 in File S1). With the exception of the locomotor activity assays, which used both the ‘summer’ and ‘winter’ simulation, the remaining experiments were all performed only under the ‘summer’ condition, as insecticide applications in the fields are more likely in conditions resembling the summer season when *D. suzukii* populations are higher and susceptible fruit are present.

**Figure 2:**
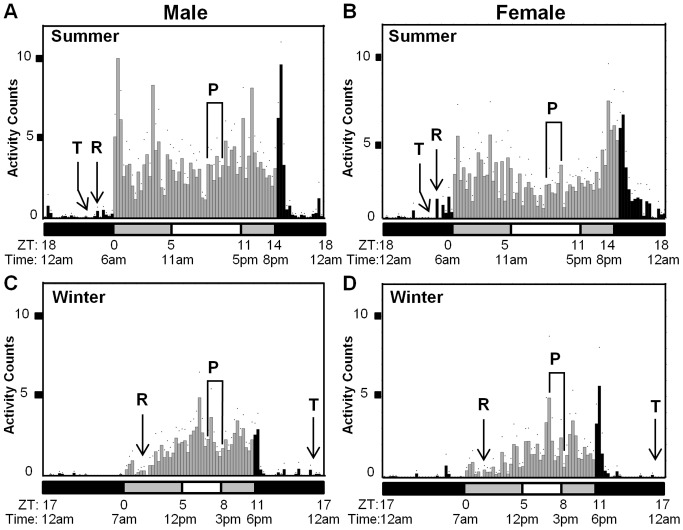
Daily locomotor activity profiles of *Drosophila suzukii* male and female flies in different seasons. Male (A and C) and virgin female (B and D) flies were subjected to temperature and light/dark cycles that simulate a typical summer (July/August) (A and B) or winter (January/February) (C and D) day in Watsonville, CA, U.S. The specific conditions for the simulation are detailed in Figure 1 and Table S1 in File S1. They were kept under these conditions for at least seven days, and their activity levels were recorded using *Drosophila* Activity Monitoring System (DAMS) (Trikinetics, Inc.). The flies were around three days old at the start of the experiments. The locomotor activity levels of individual flies were measured in 15 min bins and then averaged to obtain a representative group profile, as illustrated in eduction graphs generated using FaasX. The activity level is represented by raw activity counts (X-axis) as measured by DAMS [18]. Data shown here resulted from averaging the second to the fourth days of the recordings, generating the 24 h profiles shown in the panels. Vertical bars represent the activity recorded in 15 min bins during times when the lights were on (light gray bars) or off (black bars). Three levels of light intensity are indicated by the horizontal bars beneath the graphs. Black bars = lights off; dark gray bars = dim light; white bars = bright light. Both zeitgeber (ZT) and natural time for changes in light intensity are shown underneath the horizontal bars. For summer conditions: lights-on time (ZT0) and lights-off time (ZT14) is set at 6am and 8pm respectively. The period representing peak summer temperature, 22.2°C between 2pm to 3:30pm, is denoted by the letter “P”; the time point at which temperature reaches the trough, 12.2°C at 4am, is marked by an arrow labeled “T”; and the time point at which temperature starts to rise from the trough, at 4:30am, is marked by an arrow labeled “R”. For winter conditions: lights-on time (ZT0) and lights-off time (ZT11) is set at 7am and 6pm respectively. The period representing peak winter temperature, 16.7°C between 2pm to 3:30pm, is denoted by the letter “P”; the time point at which temperature reaches the trough, 6.8°C at 11pm, is marked by an arrow labeled “T”; and the point at which temperature starts to rise from the trough, 6.8°C at 9am, is marked by an arrow labeled “R”. 64 flies were used for each treatment at the start of the experiment. Taking into account the mortality of flies during the course of the experiment, sample sizes are as follows: Male (summer) n = 59 (92% survival); Female (summer) n = 54 (84% survival); Male (winter) n = 45 (70% survival); Female (winter) n = 21 (32% survival).

Similar to *D. melanogaster*, most of the activity of *D. suzukii* occurs during the day or lights-on period, while rest/sleep are consolidated at night during the dark period (Figure 2) [19], [54]. In general, the total activity over a circadian day is much lower for *D. suzukii*, as compared to *D. melanogaster* tested under similar conditions (unpublished, RS Kwok) making it difficult to determine the periodicity of their locomotor activity rhythm using DAMS. In addition, *D. suzukii* males appeared to be more active than females in both ‘summer’ and ‘winter’ conditions, as illustrated by overall greater activity counts over a circadian day (Figure 2). This is consistent with previous observations indicating that *D. suzukii* males are more active while females are more passive [55], as well as our field observations in California. We speculate that this is likely due to mate-finding and potential sexual competition behaviors in males.

Whereas the daily activity profiles of male and female *D. suzukii* do not differ dramatically in either of the seasonal conditions, the activity patterns of *D. suzukii* appeared quite different between ‘summer’ and ‘winter’ conditions (Figure 2). In the ‘summer’, *D. suzukii* assumed a more bimodal activity pattern, where maximum activity occurs at dawn and dusk. This was especially pronounced in females (Figure 2B), where activity peaks were preceded by anticipatory progressive increase in activity, highlighting the characteristic involvement of the circadian clock in driving this pattern. Males also showed maximum activity levels at dawn and dusk, but no clear anticipatory increase in activity was observed (Figure 2A), suggesting that the increase activity might be attributed to “startle” response as a result of change in lighting conditions [17], [56]. It is unclear whether a gradual increase in light intensity, in addition to the gradual temperature ramping used in our experiments, will result in more pronounced anticipatory responses and bimodal activity peaks in males. In the ‘winter’, overall activity levels of *D. suzukii* decreased dramatically (compare Figure 2C–D to 2A–B), and assumed a more unimodal peak around midday when the temperature and light intensity levels are around or at peak values respectively (Figure 2C–D). An increase in activity can also be observed at dusk, especially in females (Figure 2C–D), but could be due to “startle” response that is not based on circadian clock function. Not surprisingly, as ‘winter’ nighttime temperatures may not be amenable to activity, we observed higher nocturnal activity in flies of both sexes under the ‘summer’ conditions (Figure 2).

Sixty-four female and male flies were subjected to each environmental treatment, and, as expected, higher mortality was observed in the ‘winter’ simulation for both sexes. 92% of males and 84% of females survived the duration of the ‘summer’ experiment, while only 70% of males and 32% of females survived the entirety of the ‘winter’ experiment (see legend of Figure 2). Previous laboratory experiments simulating cold winter conditions in more Northern latitudes, such as Oregon and Washington, U.S., resulted in 100% *D. suzukii* adult mortality by 29 days exposure to constant 3°C with a 7-day freeze period [57]. As suggested by our results, the California climate may be more amenable to *D. suzukii* survival and winter activity, making this species a serious threat to agricultural industries in California or other regions with similar climate.

### Expression of Xenobiotic Detoxification Genes in Drosophila Suzukii vary with the Time of Day

Previous *D. melanogaster* transcriptome analysis using microarrays and RNA sequencing showed that detoxification genes are well represented in cycling genes [29]–[37], suggesting that insecticide detoxification may be regulated by the circadian clock. Studies examining the circadian expression of specific Phase I and Phase II detoxification genes have been performed in both *D. melanogaster* [45], [46], [49] and mammals [48], [58] implicating PAR-domain basic leucine zipper (PAR bZIP) transcription factors, for example *D. melanogaster* PDPD1ε, as key activators of circadian as well as inducible detoxification genes. *D. melanogaster* CncC (cap ‘n’ collar isoform-C), the ortholog of mammalian Nrf2 (NF-E2-related factor 2), has also been identified as a central transcriptional regulator of key detoxification genes including those encoding CYPs and GSTs, but it is involved in basal and inducible expression, rather than circadian control [43].

To test the hypothesis that circadian expression of insecticide detoxification genes may be the basis for *D. suzukii* chronotoxicity in response to commonly used insecticides, we assayed the expression of several Phase I and II detoxification genes over a circadian cycle. The six candidate target genes were selected based on one or a combination of the following criteria: previous observations of rhythmic expression in *D. melanogaster* (*Cyp6a2, Cyp6g1,* α*-est7*) [31], [46] *;* association with metabolic insecticide resistance in natural populations (*Cyp6g1)* [44] or when overexpressed in *D. melanogaster* (*Cyp6a2, Cyp6g1, Cyp12d1*) [59]–[61]; and inducibility of gene expression by insecticides or other xenobiotics (*Cyp6a2, Cyp6g1, Cyp12d1, GstD2, GstD7*) [61]. Previous *D. melanogaster* expression studies by microarray [31] and quantitative real-time PCR [46] using various tissues as starting materials (bodies only and whole flies) concluded that *Cyp6a2, Cyp6g1*, and α*-est7* are strongly rhythmic, with peak expression between ZT4 to ZT8 (middle of light period) with clock entrainment conditions of 12:12 h light:dark cycle and constant temperature of 25°C. Other *D. melanogaster* microarray analyses and meta-analyses that mostly used fly heads as starting materials [29], [30], [32]–[35] have also identified various other P450 *Cyp* and *Gst* genes as rhythmically expressed, but our study focused on genes that are cycling in the fly body. *Cyp12d1* has not been shown to be circadian regulated but is known to be only 1 of 89 P450 *Cyp* genes that were tested by Willoughby et al. [61] to be induced by DDT (organochlorine), and conferred resistance to DDT and dicyclanil (insect growth regulator) when overexpressed in *D. melanogaster* [59]. Even though *GstD2* and *GstD7* are not known to be rhythmically expressed over the circadian day, their paralog *GstD1* has been shown to be circadianly regulated in *D. melanogaster* [31], [46]. The other reason they were chosen for this analysis was that while *GstD2* represented the only *Gst* gene out of 37 tested that was inducible by DDT [61], transcription of both *GstD2* and *GstD7* were induced by the known metabolic enzyme inducer phenobarbital [61].

**Figure 3:**
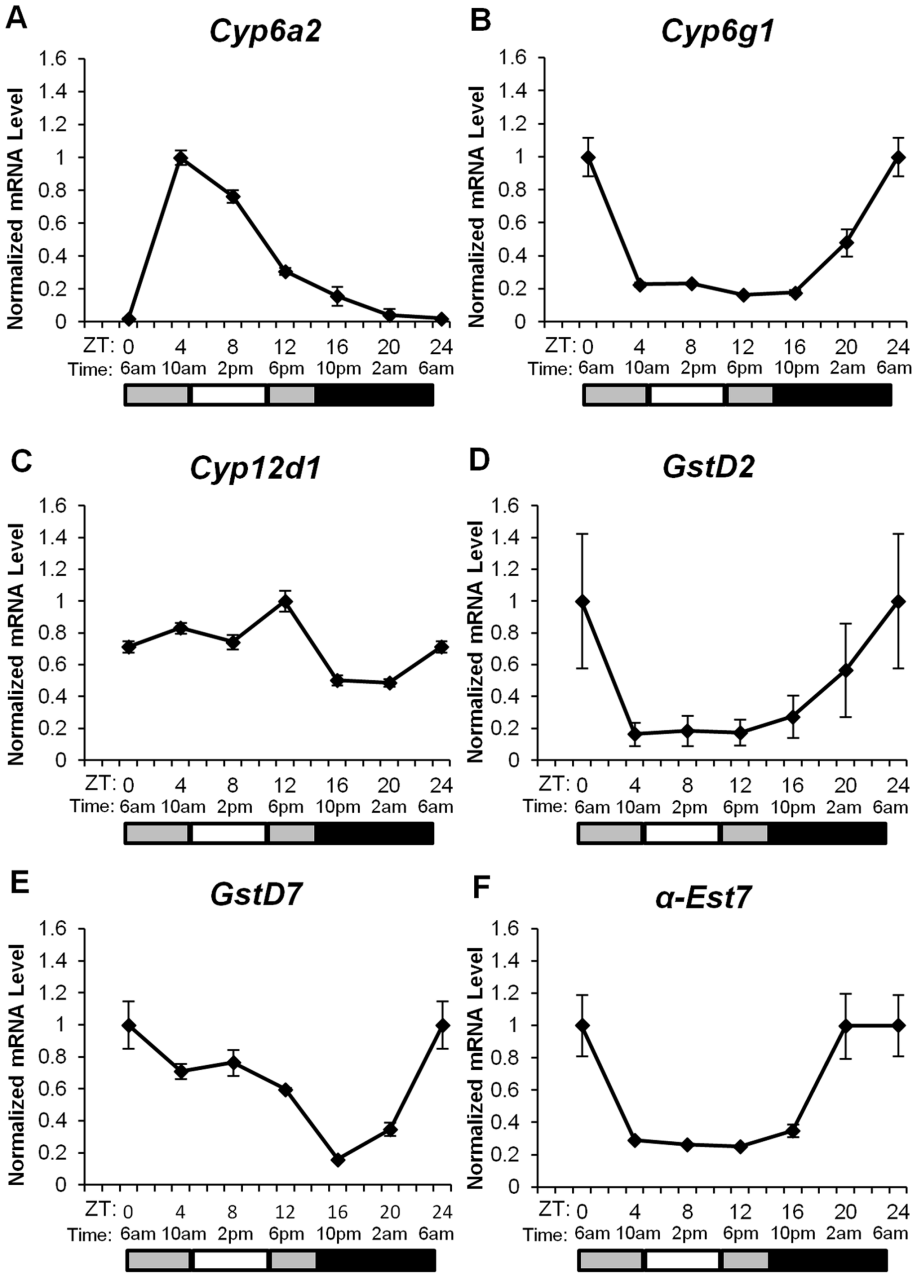
Daily mRNA expression profiles of three classes of detoxification genes. Transcript levels for three cytochrome P450 genes (A, B, C), two glutathione S-transferases (D and E), and an *α-esterase 7* (F) were assayed. RNA was extracted from a mixed population of adult male and female flies that were subjected to entrainment conditions approximating Watsonville, CA, U.S. summer (July/August) temperature and light/dark conditions (see Figure 1 and Table S1 in File S1) for three days, and collected via freezing with dry ice on the fourth day. Flies were collected at 6 time points (ZT0, 4, 8, 12, 16, and 20) throughout the circadian day, as indicated on the X-axis. ZT0, which is equal to 6am, is also plotted as ZT24 to represent both the beginning and the end of a circadian day. Horizontal bars beneath the X-axis show the light conditions present during the corresponding time of day. Black bars = lights off; dark gray bars = dim light; white bars = bright light (see Figure 1 and Table S1 in File S1). Relative levels of mRNA were quantified by real-time PCR and normalized to *Cbp20* expression. Graphs are scaled with the time of highest gene expression set to 1. Each point represents the average of three biological replicates (n = 3).

Previous studies examining the rhythmic expression of some of our target genes were performed using *D. melanogaster* that were entrained in 12:12 h light:dark cycle at a constant temperature of 25°C. This is the first study to examine circadian expression of detoxification genes of *D. suzukii* entrained in light and temperature conditions that approximate that of the location in which the flies were first collected in North America near Watsonville. We used the ‘summer’ condition for entrainment of our flies before RNA collection for reasons mentioned earlier. This entrainment program has a longer photoperiod (14:10 h light:dark) and lower temperature range (12.2°C to 22.2°C) than the standard test conditions in *D. melanogaster* gene expression analysis. As a result, even if we assume there are no species-specific differences between circadian regulation of genes such as *pdp1*ε, a PAR bZIP transcription factor which has been implicated to control expression of detoxification genes in *D. melanogaster* [46], and effector detoxification genes, we would expect differences in the phase and amplitude of the cycling genes. Out of the six genes we tested, five of them appeared to be rhythmic (Figure 3). *Cyp12d1* has not been shown previously to be clock-regulated in other *Drosophila* species, and is the only gene out of the six that did not show consistent cycling (Figure 3C). *GstD2* and *GstD7* have not been shown in *D. melanogaster* heads to be rhythmically expressed over a circadian day, but appeared to be cycling in *D. suzukii* fly bodies with a similar phase, with expression peaking at ZT0 (Figure 3). All three genes that were shown to be circadianly regulated in *D. melanogaster, Cyp6a2, Cyp6g1,* and *α-esterase 7* [46], showed strong cycling in *D. suzukii* as well (Figure 3A–B and F). Whereas the peak expression of *Cyp6g1* and *α-esterase 7* is at ZT0, the peak expression of *Cyp6a2* was phase-delayed by 4 hours, indicating that perhaps the expression of *Cyp6a2* is activated through a different mechanism.

Based on studies of the *D. melanogaster* circadian clock, the peak mRNA expression of target genes that are directly regulated by CLOCK (CLK), the key transcriptional activator of clock-controlled genes, generally coincides with the evening activity peak [15]. *Pdp1*ε, a transcriptional activator of many circadian output genes, is one such target [62], [63]. We therefore expected the peak expression of *D. suzukii Pdp1*ε to be around ZT14 in the ‘summer’ entrainment condition. If indeed the expression of the detoxification genes we tested are directly activated by PDP1ε protein, as suggested to be the case in *D. melanogaster* [46], then we might expect their peak expression to be shortly after ZT14. However, most of our cycling genes did not reach their peak expression until about 10 hours later at ZT24 (or ZT0) (Figure 3). We speculate that perhaps the detoxification genes tested in this study are not direct transcriptional targets of PDP1ε protein. It is possible that other transcription factors might be involved as intermediate regulators to create the lag time between the peak expression of *Pdp1*ε and the detoxification genes tested here. Alternatively, there may be other mechanisms that generated this time lag. For example, *clock (clk)* is a direct target gene of *Pdp1*ε, but the peak expression of the *clk* gene is roughly 12 hours after that of *Pdp1*ε due to the function of a transcriptional repressor VRILLE (VRI) that counteracts the activation of *clk* by PDP1ε [62], [63].

### Circadian Variation in Drosophila Suzukii Susceptibility to Acute Exposure of Insecticides

Acute toxicity experiments were independently performed on at least two different days for all doses of malathion with at least 64 (sum of flies from the three replicate vials) total flies per circadian time point and at doses ranging from 0.00 to 0.54% of the California strawberry label rate (0.0692 µmol AI/cm^2^) (Table 1). For fenpropathrin, at least 3 independent treatment days were performed for all doses ranging from 0.00 to 7.86% of the field rate (0.0117 µmol AI/cm^2^), with at least 103 total flies (sum of flies from the three replicate vials) per time point (Table 1).

Variability between time points (i.e. potential violation of the assumption of homogeneity of variance) and within doses (i.e. potential over dispersion of the model) was a concern for our analysis. As we chose to use formulated product and a *D. suzukii* population with heterogeneous genetic background to more closely mimic field conditions, our data were more variable than might be expected in studies that used technical grade active ingredient and isogenic fly lines (eg. [45]). This presents a challenge for fitting dose-response curves as it may artificially inflate the p-values. A mixed model logistic regression was performed in SAS 9.2 (SAS Institute 2008) to account for variability and ensure that time point had a significant effect. Once this was verified, fitting of dose-response curves and calculation of LC_50_ was performed using PRISM v.5.0 because it is more biologically relevant and provides intuitive graphs and LC_50_ readout.

**Figure 4:**
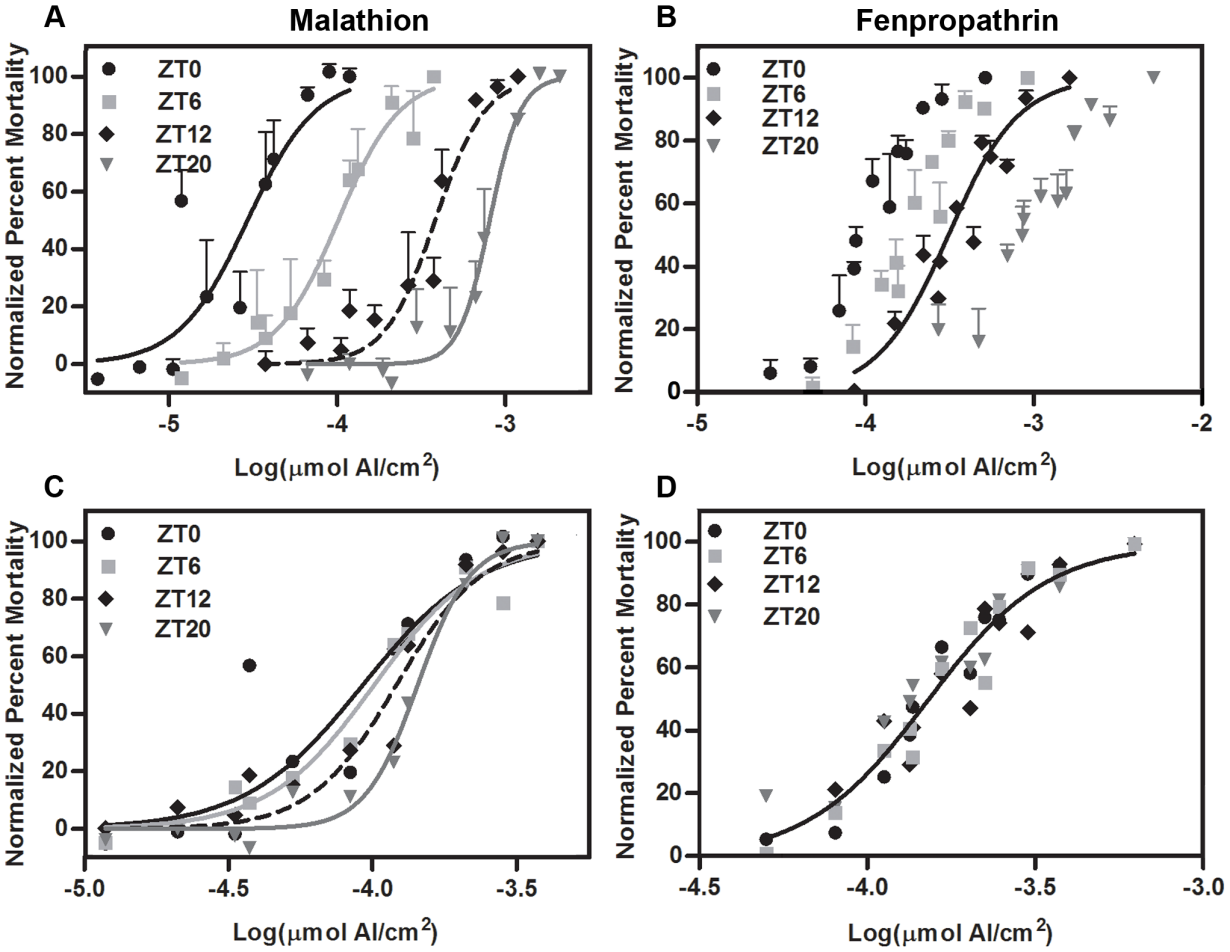
Chronotoxicity of female *Drosophila suzukii* for acute contact exposure to insecticides. Normalized percent mortality (Y-axis) of female *D. suzkii* exposed to malathion (A,C) and fenpropathrin (B,D) for 1 h (±5min) was assessed at 4 time points (ZT0, 6, 12, 20) for flies entrained to conditions approximating Watsonville, CA, U.S.A. summer (July/August) temperature and light/dark conditions (see Figure 1 and Table S1 in File S1) for approximately 50 h prior to exposure. The insecticide dose [expressed in Log(µmol of insecticide active ingredient (AI)/cm^2^) (X-axis)] was prepared from formulated insecticide using acetone as a solvent 1 h prior to the contact experiment and 250 µL was applied to coat the interior of a 20 mL scintillation vial (56.9 cm^2^). Flies were held in pesticide-free diet at Watsonville, CA summer conditions for 72 h (±5min) after exposure when mortality was assessed as a count of dead and moribund flies combined. Points on the graph represent the mean normalized percent mortality exhibited at each Log(Dose) for the female flies assayed (across the three replicate vials) (Table 1). The points were separated along the x-axis using the “nudging” function in PRISM v.5.0 (GraphPad Software, Inc.) to prevent overlap of the standard error bars (i.e. the standard error of the mean of the normalized percent mortality across the three replicates) (A, B). The variable slope sigmoidal dose response best-fit curves produced in PRISM v.5.0 for malathion are graphed and similarly separated along the x-axis with the points (A), and the single best-fit curved produced for all time points for fenpropathrin was shifted toward the middle of the graph, again using the “nudging” function in PRISM v.5.0 (B). The points and curves are presented without their error bars and without shifting along the x-axis for dose response comparison of malathion (C) and fenpropathrin (D).

The normalized variable slope sigmoidal dose response curve fit was poorest for ZT0 (R^2^ = 0.7305), which was the time point that exhibited the highest variability in mortality response and the lowest LC_50_ (Figure 4A, 4C, and Table 2). Although the 95% confidence interval for the LC_50_ estimates overlap for malathion at most time points, a comparison of the slope and LC_50_ estimates together for each time points reveals a significant difference between the dose response curves at the different Zeitgeber time points (*F*_6,124_ = 3.52, *P* = 0.0030) (Figure 4C and Table 2). Female *D. suzukii* were most tolerant of contact with malathion at ZT20 (LC_50_ (95% CI): 1.44E-4 (1.29E-4–1.60E-4)), and least tolerant at ZT0 (LC_50_ (95% CI): 9.25E-5 (7.15E-5–1.20E-4) (Figure 4A, 4C, and Table 2). For fenpropathrin, the dose response curve fit was poorest at ZT20 (R^2^ = 0.8295) (Table 2). The LC_50_ estimates for each time point fall within the 95% confidence intervals for all of the time points, and indeed there is no difference between the dose response curves at the different Zeitgeber time points (*F*_6,136_ = 2.04, *P* = 0.0644), therefore a single dose response curve was fit (Figure 4B, 4D, and Table 2).

## Discussion

### A First Look at Locomotor Activity Rhythms of Drosophila Suzukii under “Natural” Conditions

For *D. suzukii* entrained to ‘summer’ conditions (14:10 h light:dark cycle; Max 22.2°C/Min 12.2°C), we observed a bimodal pattern of activity that was similar to results observed in laboratory *D. melanogaster* locomotor assays, with an afternoon ‘siesta’ during the period with the highest light intensity and warmest temperature. However, under ‘winter’ conditions with a shorter photoperiod and colder temperature range (11:13 h light:dark cycle; Max 16.7°C/Min 6.8°C), activity profile of *D. suzukii* was more unimodal, with an activity peak in the middle of the day during the time of highest light intensity and temperature.

Based on a large body of work examining the molecular mechanisms underlying light and temperature entrainment of the *D. melanogaster* circadian clock, it is well accepted that both temperature and light are zeitgebers that are sufficient to entrain the circadian clock independently, and the integration of the two entrainment pathways coordinate with and reinforce each other to regulate activity rhythms (reviewed in [64], [65], [66]). This is intuitive because in nature, shorter day length in the winter is usually accompanied by colder temperatures while longer day length in the summer is associated with warmer temperatures. Light entrainment of the clock primarily functions through the transcription factor TIMELESS (TIM) [67], [68] that senses light input via interaction with the circadian photoreceptor CRYTOCHROME (CRY) [69]. TIM forms a complex with another circadian transcription factor PERIOD (PER) at night-time, and together they repress the expression of the circadian transcriptome (reviewed in 66). In the presence of light, TIM protein undergoes proteasome-dependent degradation, thereby entraining the clock to light cues [70], [71]. In the context of activity levels, degradation of TIM and consequently PER generally leads to increase in activity, thereby restricting most activity during the daytime. More recent studies that used more natural conditions to study *D. melanogaster* clock-regulated activity rhythms suggested that temperature, rather than light, might actually be the more critical zeitgeber for entrainment in nature [19], [24]. Temperature entrainment of the clock has been shown in laboratory studies to partly depend on the thermosensitive splicing of a 3′-terminal intron *dmpi8* (*Drosophila melanogaster period* intron 8) in *per* that is dependent on the function of the phospholipase C NORPA [72], [73]. This mechanism has been proposed to be involved in seasonal adaptations of the clock. For example, in the winter morning, the colder temperature increases the splicing efficiency of this specific intron, leading to an earlier upswing of *per* mRNA levels and consequently PER proteins, as compared to a summer morning. This results in phase advance of clock-controlled evening activity onset that will otherwise takes place near dusk, ensuring that flies are active during the afternoon instead of early evening, when it is much colder and not as amenable to activity. This explains why activity profiles of flies are generally more unimodal in colder temperatures [72], similar to what we observed in *D. suzukii* in the ‘winter’ conditions (Figure 2C–D). However, the importance of *per dmpi8* splicing as a mechanism to control evening activity onset in more natural conditions has been in question recently, as both wild type flies and *per^0^* mutants show the same linear relationship between time of evening activity onset and temperature over a range from 7°C to 30°C [19], indicating that other mechanisms might be involved. A second temperature entrainment pathway of the clock is believed to act through NOCTE, a glutamine rich protein with yet unknown function [25]. Since natural entrainment requires both light and temperature zeitgebers, it is not surprising that there are mechanistic interactions between the light and temperature entrainment pathways that provide robustness to the system [26]. For example, light can rapidly increase *tim* mRNA levels in early morning, but only at cold temperature [53]. Again, this mechanism serves to ensure advancing of evening activity in the winter seasons.

As we noted earlier, the temperatures used in our studies, which resemble actual temperature ranges in Watsonville, CA, U.S.A., are much lower than what have been used previously in most *D. melanogaster* laboratory studies to elucidate temperature entrainment mechanisms (reviewed in [65]). It has been proposed that when temperature is below 15°C, temperature may be dominant over light as zeitgeber, since *D. melanogaster* flies can remain rhythmic in constant light conditions, but only in temperatures below 15°C [74]. Light induces TIM degradation and constant light usually leads to arrhythmicity due to persistent low level of this key circadian transcription factor. The fact that flies remain rhythmic below 15°C suggests an inability to sense light effectively. Together with the recent proposal by Vanin et al. [19] and Currie et al. [24] that temperature might be the more critical zeitgeber in nature, we therefore propose that temperature may be the dominant zeitgeber in the ‘winter’ condition for our *D. suzukii* activity assays. Assuming thermosensitive splicing of the intron in the *D. suzukii per* ortholog that corresponds to *dmpi8* is partly responsible for timing evening activity, *per* splicing is expected to be more efficient in the ‘winter’ conditions, allowing the ‘evening’ activity peak that normally occurs near dusk at warmer temperatures to phase advance, creating a more unimodal activity profile. This makes ecological sense as activity at lower temperatures towards the latter part of the circadian cycle will be more costly energetically. As mentioned previously, other mechanisms in addition to splicing of *per dmpi8* may be at work to control timing of evening activity onset. A study in *D. melanogaster,* which examined how temperature cycles entrain the endogenous clock, determined that peak temperature and length of thermophase, i.e. duration of cycle when temperature is high, are key factors that determine the timing of peak activity levels in the absence of light [24]. Under total darkness, temperature gradient cycles alone can entrain the endogenous clock over a wide range of temperatures, and the timing of peak activity closely tracked the temperature peak. This phenomenon is consistent in conditions where the thermophase is either short or long. Interestingly, while the flies showed unimodal activity patterns when the thermophase is short, a long thermophase transformed their activity profile to bimodal, where the first activity peak occurs at the time of initial temperature ascent from trough level, and the second activity peak tracked the mid point of the long thermophase. These observations can be extrapolated to explain the unimodal and bimodal nature of the observed *D. suzukii* activity patterns, again highlighting the strong contribution of temperature as a determinant for this aspect of *D. suzukii* activity profile. In the ‘winter’ simulation, the time when temperature starts rising from trough level (labeled as “R” in Figure 2C–D) to the time when it reaches the trough again (labeled as “T” in Figure 2C–D) lasts 15 hours, and can be considered to have a short thermophase, as compared to the corresponding period in the ‘summer’ simulation (between “R” and “T” in Figure 2A–B), which takes the full 24 hour cycle. As Currie et al. [24] have shown, temperature also appeared to play an important role in determining the timing of peak activity, as the strongest activity peak of *D. suzukii* flies in the ‘winter’ simulation (temperature range: 6.8°C to 16.7°C), with the exception of the startled response, occurred at or around the temperature peak (Figure 2C–D). Even though we propose that temperature is the dominant zeitgeber in the ‘winter’ simulation based on previous findings in *D. melanogaster*, the activity peak of ‘winter’ *D. suzukii* flies also coincides with period of highest light intensity, indicating perhaps the two zeitgebers reinforce each other. Future experiments will be necessary to characterize possible interaction between light and temperature at these low temperature ranges, and examine mechanisms of temperature entrainment not related to thermosensitive splicing of *per dmpi8*.

In the ‘summer’ simulation (temperature range: 12.2°C to 22.2°C) when the temperatures are warmer, *D. suzukii* has a bimodal activity profile (Figure 2A–B), which can partly be attributed by the longer thermophase. At these warmer temperatures, splicing efficiency of the intron in the *D. suzukii per* ortholog that corresponds to *dmpi8* may be lower than that in the ‘winter’ condition, resulting in less phase advance of the evening activity. Night-time temperatures are relatively milder during the summer and more amenable to activity, hence the increased nocturnal activity (Figure 2). Since the timing of the activity peaks in the warmer temperature range of our ‘summer’ simulation did not correspond to the pattern that would be predicted if temperature gradient cycles alone were used for entrainment [24], i.e. the second activity peak did not track the temperature peak (labeled as ‘P’ in Figure 2A–B), we conclude that light is a much stronger zeitgeber in the ‘summer’ condition as compared to the ‘winter’ condition, even though the temperature range used in our experiments were still quite low, going down to 12.2°C in early morning (Figure 1 and Table S1 in File S1). As a matter of fact, the temperature peak in the ‘summer’ condition coincides with a mild ‘siesta’ (Figure 2A–B) that will likely be more pronounced if higher temperature were used. It is believed that the adaptive function of the ‘siesta’ is to avoid the hot mid-day sun and dehydration [64].

Although we attempted to closely mimic natural conditions for our locomotor activity assays, we did not observe the afternoon activity peak as observed in Vanin et al. [19]. This discrepancy is likely due to the fact that the temperature range used in our experiments, even for the ‘summer’ simulation (Max/Min 22.2°C/12.2°C), is on the lower end of the temperature spectrum used in those experiments, and the occurrence of ‘afternoon’ activity peak is found to decrease dramatically at lower temperatures [19]. Another reason could be species-specific differences in activity levels and patterns. Lastly, it is unclear whether a more gradual change in light intensity could produce an afternoon activity peak, at least in the ‘summer’ simulation.

### Correlating Insecticide Chronotoxicity and Chronobiology of Drosophila Suzukii

The phenomenon of chronotoxicity was first discovered in German cockroaches *Blattella germanica* (L.) (Dictyoptera:Blattellidae) dosed with potassium cyanide [75]. Soon after, cyclic variability in susceptibility to methyl parathion (organophosphate insecticide), with the lowest mortality occurring at dawn and reoccurring every 6 hours in the boll weevil, was seen in *Anthonomus grandis* Boh. (Coleoptera:Curculionidae) [76]. Circadian variation in pesticide susceptibility has since been observed in many arthropods including *D. melanogaster* and *Apis mellifera* L. (Hymenoptera:Apidae) to multiple classes of insecticide [45], [47], [77], [78].

No difference in chronotoxicity to *D. suzukii* was observed for fenpropathrin in this study, which is consistent with results from *D. melanogaster* exposed to another pyrethroid, deltamethrin [45]. *D. suzukii* entrained at a photoperiod and temperature regime that estimates Watsonville, CA in the summer showed the highest and lowest insecticide tolerance to malathion at ZT20 (2am) and ZT0 (6am) respectively. This is not consistent with observed chronotoxicity of malathion to *D. melanogaster* maintained at 12:12 h light:dark cycle and constant 25°C, for which the highest and lowest insecticide tolerance was seen at ZT4 and ZT16 respectively [45]. We speculate that the difference in test species as well as drastically different entrainment conditions likely contributed to the difference in observations. We want to point out that the phase of peak expression for some common detoxification genes tested in our studies and the study by Hooven et al. [45] were also different, likely for the same reason. Previous chronotoxicity work has suggested that time of greatest insecticide susceptibility may correspond to the onset of a time of increased activity in the insect [12] and expression of detoxification enzymes has also been proposed as an alternate explanation for varying insecticide susceptibility over a circadian cycle [47], [76].

**Figure 5:**
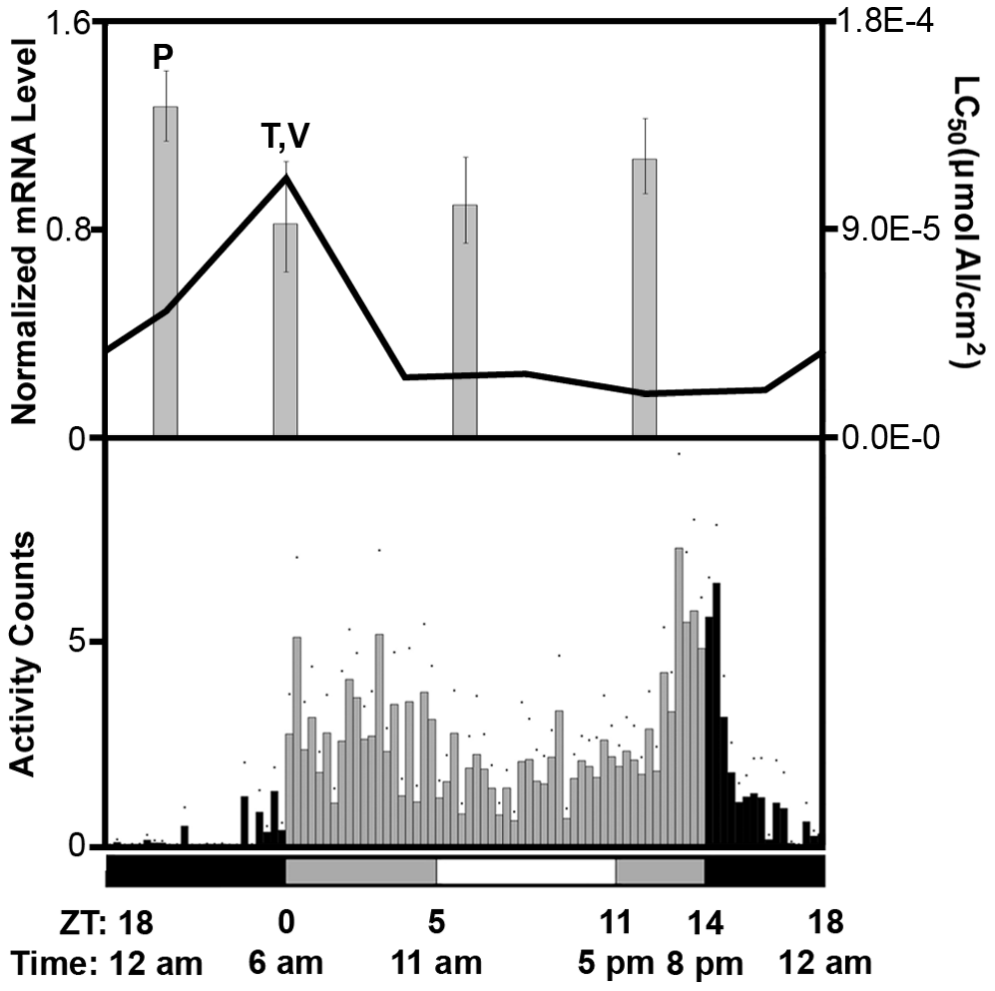
Correlating chronobiology and malathion chronotoxicity of *Drosophila suzukii.* The results from (1) daily locomotor activity assays of *D. suzukii* females (bar graph in bottom panel); (2) circadian gene expression analysis of *Cyp6g1* (line graph in top panel, left Y-axis); and (3) malathion acute toxicity assay (bar graph in top panel, right Y-axis), were compiled for side-by-side comparison and correlation. All experiments shown here were conducted in experimental condition simulating an average summer day of Watsonville, CA, U.S. (Figure 1 and Table S1 in File S1). Three levels of light intensity are indicated by the horizontal bars beneath the graph. Black bars = lights off; dark gray bars = dim light; white bars = bright light. Both zeitgeber (ZT) and natural time for changes in light intensity are shown underneath the horizontal bars. For summer conditions: lights-on time (ZT0) and lights-off time (ZT14) is set at 6am and 8pm respectively. Activity data shown here is for female flies and is the same as presented in Figure 2B. Circadian expression pattern of *Cyp6g1* in fly bodies is presented here as a representative pattern for most of the genes tested, and is the same as presented in Figure 3B, except with a shift in the X-axis to allow for easier comparison. Malathion acute 1 h LC_50_ for female *D. suzukii* was calculated using normalized variable slope sigmoidal dose response best-fit curves in PRISM v.5.0 (GraphPad Software, Inc.) at 4 time points (ZT0, 6, 12, 20) using 2–3 day old female *D. suzukii* and 11 doses of malathion, and is plotted using data from Table 2. Error bars represent the 95% confidence interval for each LC_50_. The time point with peak malathion tolerance “P”, with trough tolerance “T”, and with the most variable tolerance “V” is marked.

In this study, we saw a peak in *D. suzukii* malathion tolerance (least susceptible) at ZT20 (2am), which corresponds to the period of lowest locomotor activity and rapidly rising detoxification gene expression (*Cyp6g1, GstD2, GstD7*, and *α-esterase 7*) (Figure 5). When flies are less active, they may be receiving a lower dose of insecticide, and the moderate and rising levels of detoxification enzyme expression may help mitigate the toxicity of the insecticide they do receive. However, the fact that ZT12 (6pm) exhibits the highest *D. suzukii* activity and has an LC_50_ most similar to ZT20 (2am), when *D. suzukii* is most tolerant to malathion and the least active (Figure 5) suggests that expression profile of detoxification genes may represent a stronger determinant as compared to activity level for insecticide chronotoxicity. At ZT0 (6am), a peak in expression is observed in most of the assayed genes (*Cyp6g1, GstD2, GstD7,* and *α-esterase 7*), and this corresponds with the most variable mortality response to malathion and moderate fly activity (Figure 3 and 5). Indeed, the 95% confidence interval of the LC_50_ for malathion at ZT0 (6am) has the poorest curve fit (Figure 4 and Table 2). At time points when detoxification enzyme titers are high and play a role in survivorship of the test subjects, it is expected that the mixed genetic background of the flies utilized in this study may show variable phenotypic response and contribute to increased variability in mortality. Similarly, an increase in variability in developmental time is seen when *D. suzukii* are placed on poorer quality host fruits (D Bellamy et al., unpublished data). Again it is speculated that phenotypic variation due to variable genetic background between test subjects is more obvious in a context where there is strong selection pressure playing a role in survivorship (D Bellamy et al., unpublished data). It is also important to note that the initial step of malathion metabolism by oxidative sulfuration, which can be attributed to the activity of cytochrome P450s, actually converts malathion into the more toxic intermediate malaoxon, before it is further detoxified by GSTs and esterases [79], [80]. Therefore, the peak in *Cyp6g1* expression at ZT0 may partly explain the trough in malathion tolerance observed at ZT0 (Figure 3B and 5), especially if enzyme activity of CYP6G1 is responsible for bioactivation of malathion (i.e. conversion malathion to the more toxic malaoxon). The initial competing influence of enzyme activities encoded by *Cyp*s, *Gsts*, and *esterases*, with respect to malathion toxicity, likely contributed to the variability of mortality observed at ZT0 as well. ZT0 exhibits moderate *D. suzukii* activity and only slightly higher activity as compared to ZT6 (12pm), which corresponds to the beginning of the prolonged trough period of expression for most detoxification genes tested in this study, except for *Cyp6a2* whose expression level is still close to its peak (Figure 3A and 5). High activity of CYP6A2 may contribute to the bioactivation of malathion at ZT6, keeping malathion tolerance at a low level. We conclude that circadian gene expression of detoxification genes strongly contributes to the chronotoxicity of *D. suzukii* in response to malathion. On the contrary, periods of higher *D. suzukii* activity, at least in laboratory conditions, may not correspond to periods of higher insecticide susceptibility. Future experiments may be performed to investigate if the circadian phases of enzyme activities encoded by *Cyp* and *Gst* genes is tightly linked to phases of gene expression. However, this is likely complicated by the difficulty of isolating enzyme activity involved in insecticide detoxification, which may be encoded by only a fraction of the large number of *Cyp* and *Gst* genes. In addition to activity levels and expression of detoxification genes, there are likely other factors that might contribute to chronotoxicity. For example, the level of endogenous glutathione, an antioxidant used by GST enzymes for detoxification, has been shown to vary over the circadian cycle [49].

### Implications for Pest Management of *D. suzukii*

The majority of organic berry farm acreage is concentrated on the west coast of the U.S., and now exceeds 1500 total farms [11], [81]. A growing industry, organic berry growers face a complex management problem with *D. suzukii.* The organic berry community has suggested vacuuming, mass bait and kill trapping, and microbial control as potential control tactics (Pers. Comm., Mark Bolda). In particular, the efficacy of vacuuming will be improved by timing for peaks in *D. suzukii* activity. Vacuuming for control of *Lygus hesperus* (Knight) in organic strawberries is a common control tactic [82], and better removal of the bugs from the plants occurs when the *Lygus lineolaris* P. de. B. are active higher in the strawberry canopy [83]. In a circadian comparison of sampling techniques for *L. lineolaris* adults and nymphs, chronobiology impacted sampling efficacy and peak captures varied by technique and time of day [84]. Optimization of application timing (peak activity and within production season) would be expected to improve the efficacy of microbial control [85], taking into account the environmental restrictions of the microbial agent (e.g. humidity and temperature) [86].

The laboratory reared *D. suzukii* used for these experiments were highly susceptible to these insecticides. The LC_50_ for *D. suzukii* acute (1 h) contact exposure to malathion was less that 0.21% of the field rate at all time points, and was less than 1.58% of the field rate for fenpropathrin. The difference between the time point of most susceptibility to malathion from that of least susceptibility is 0.07% of the field rate. Adult *D. suzukii* experience 100% contact toxicity after direct spray of malathion at field rate in the lab [11]. After field application of malathion at field rate, adult *D. suzukii* captured in a sweep net remain significantly lower than the control for up to 10 days post-treatment [11]. Timing the field contact application for a time of peak susceptibility would not be expected to significantly improve malathion field efficacy for fly populations that are susceptible. Chronotoxicity may provide valuable information if field *D. suzukii* populations begin to show tolerance to malathion via upregulation of detoxification enzymes. Any improvement in pesticide efficacy may allow for application of lower rate of insecticide, which can mitigate concerns for insecticide residue in food and the environment. For example, insecticide maximum residue level in cherries for export has become a concern due to insecticide usage for control of *D. suzukii* [87]. Finally, optimizing spray timing for chronotoxicity may improve efficacy of marginally toxic products.

Pyrethrin (Pyganic® EC 5.0, McLaughlin Gormley King Co., Minneapolis, MN) contact toxicity at field rate in the lab by direct spray results in 80% *D. suzukii* adult mortality [11]. After field application of pyrethrin at field rate, adult *D. suzukii* captured in a sweep net remains significantly different than the control for only 1 day post treatment (evaluations were made at 1, 5, 10, and 14 days post treatment), and the study concludes that there is no residual control of *D. suzukii* in the field [11]. Pyrethrin is one of two insecticide options for *D. suzukii* control in organic production settings [11], and even small increases in efficacy would be beneficial to growers. Pyrethrins are the natural product from which the synthetically-produced pyrethroids are derived [38]; therefore, there may be no significant difference in chronotoxicity for pyrethrins. On the other hand, in field situations, pest activity plays a larger role in insecticide efficacy than in laboratory studies due to increased feasibility of insecticide avoidance behavior. This is especially true in medically important insect disease vectors, e.g. mosquitoes, as it is common to expect increased efficacy of insecticides with increased activity of the insect, and changes have been observed in spatio-temporal biting patterns based on insecticide usage [88]–[90]. In reality, EPA regulations, weather, activity of beneficial insects, harvest schedules, and availability of machinery and labor tend to dictate the time of day that growers perform field applications. Photostability of the insecticide and effect of temperature on the toxicological effects of the insecticide are also important contributors to field toxicity [38], [91], [92]. A field trial with a marginal product would be a potentially interesting avenue for future research in this topic.

Current *D. suzukii* management strategies rely heavily on insecticide usage, because other pest management tactics are still being developed and optimized (e.g. monitoring strategies and cultural controls) [3], [6], [10], [11], [93]. This paper presents the first look at *D. suzukii* chronobiology, chronotoxicity, and detoxification gene expression. Chronobiology of *D. suzukii* provides valuable insights for both monitoring and control efforts, as insect activity is a crucial consideration in many pest management tactics. Further field studies are necessary for the application of *D. suzukii* chronotoxicity to field management practices; however, this information may help improve efficacy of insecticide applications.

## Supporting Information

File S1 **Materials and Methods,** Primer sequences for quantitative real-time PCR gene expression analysis. **Table S1,** Percival environmental chamber programs approximating Watsonville, CA, U.S.A.
(DOCX)
