# Supplementary material for: Integrating Circadian Activity and Gene Expression Profiles to Predict Chronotoxicity of Drosophila suzukii Response to Insecticides
Source: PLoS One. 2013 Jul 5;8(7):e68472. doi: 10.1371/journal.pone.0068472 (PMC3702611; doi:10.1371/journal.pone.0068472)
Supplement: File S1 — Materials and Methods, Primer sequences for quantitative real-time PCR gene expression analysis. Table S1, Percival environmental chamber programs approximating Watsonville, CA, U.S.A. (DOCX) [file pone.0068472.s001.docx]

**Supporting Information**

Materials and Methods S1:

*Primer sequences for quantative real-time PCR gene expression analysis*

Primers for amplifying *Cbp20*

F 5’- AGGAGCAGATCCACGAGCTC -3’

R 5’- ACTCCACGAAGCAGAAGCC -3’

Primers for amplifying *Cyp6a2*

F 5’- GAGACCTCCTCCTCGAC -3’

R 5’- TGAGATGACCTGGTTCAAGTAG -3’

Primers for amplifying *Cyp6g1*

F 5’- GGTCCAGGAAACCACAGC -3’

R 5’- ACAGCCTCGTCCTTGACC -3’

Primers for amplifying *Cyp12d1*

F 5’- TGGTTAAGGGATCCTGAGACGG -3’

R 5’- TGGCATCCCGATTGAACTCCAC -3’

Primers for amplifying *GstD2*

F 5’- ACGGTCATCATGGTGGCC 3’

R 5’- TCCTTGCCGTACTTCTCCAC 3’

Primers for amplifying *GstD7*

F 5’- AAGGCTTTGGGTCTGGAGCTG -3’

R 5’- TGCCGTACTTCTCCACCAGATAG -3’

Primers for amplifying *α-Esterase-7*

F 5’- TGTTGGCCCTCAAGTGGATCAAG -3’

R 5’- TGGCACTGCCCGACTGCAAG -3’

Table S1. Percival environmental chamber programs approximating Watsonville, CA, U.S.A. summer (July/August July/August 14:10 L:D, Max: 22.2°C, Min: 12.2°C) and winter (January/February 11:13 L:D Max: 16.7°C, Min: 3.3°C) temperature and light/dark conditions.

| **Summer** | | | | **Winter** | | | |
| --- | --- | --- | --- | --- | --- | --- | --- |
| **Lights** | **ZT** | **Time** | **°C** | **Lights** | **ZT** | **Time** | **°C** |
| 1 | 0 | 6:00 AM | 16.7 | 0 | 23 | 6:00 AM | 6.8 |
|  |  | 6:30 AM | 17.5 |  |  | 6:30 AM | 6.8 |
| 1 | 1 | 7:00 AM | 18.0 | 1 | 0 | 7:00 AM | 6.8 |
|  |  | 7:30 AM | 18.5 |  |  | 7:30 AM | 6.8 |
| 1 | 2 | 8:00 AM | 18.9 | 1 | 1 | 8:00 AM | 6.8 |
|  |  | 8:30 AM | 19.1 |  |  | 8:30 AM | 7.0 |
| 1 | 3 | 9:00 AM | 19.4 | 1 | 2 | 9:00 AM | 8.3 |
|  |  | 9:30 AM | 19.7 |  |  | 9:30 AM | 9.7 |
| 1 | 4 | 10:00 AM | 20.0 | 1 | 3 | 10:00 AM | 11.1 |
|  |  | 10:30 AM | 20.3 |  |  | 10:30 AM | 12.2 |
| 2 | 5 | 11:00 AM | 20.6 | 1 | 4 | 11:00 AM | 13.3 |
|  |  | 11:30 AM | 20.9 |  |  | 11:30 AM | 14.2 |
| 2 | 6 | 12:00 PM | 21.1 | 2 | 5 | 12:00 PM | 15.0 |
|  |  | 12:30 PM | 21.3 |  |  | 12:30 PM | 15.6 |
| 2 | 7 | 1:00 PM | 21.6 | 2 | 6 | 1:00 PM | 16.1 |
|  |  | 1:30 PM | 21.9 |  |  | 1:30 PM | 16.4 |
| 2 | 8 | 2:00 PM | 22.2 | 2 | 7 | 2:00 PM | 16.7 |
|  |  | 2:30 PM | 22.2 |  |  | 2:30 PM | 16.7 |
| 2 | 9 | 3:00 PM | 22.2 | 1 | 8 | 3:00 PM | 16.7 |
|  |  | 3:30 PM | 21.9 |  |  | 3:30 PM | 16.4 |
| 2 | 10 | 4:00 PM | 21.6 | 1 | 9 | 4:00 PM | 16.1 |
|  |  | 4:30 PM | 21.3 |  |  | 4:30 PM | 15.3 |
| 1 | 11 | 5:00 PM | 21.1 | 1 | 10 | 5:00 PM | 14.4 |
|  |  | 5:30 PM | 20.5 |  |  | 5:30 PM | 13.1 |
| 1 | 12 | 6:00 PM | 19.4 | 0 | 11 | 6:00 PM | 11.7 |
|  |  | 6:30 PM | 18.5 |  |  | 6:30 PM | 11.2 |
| 1 | 13 | 7:00 PM | 17.8 | 0 | 12 | 7:00 PM | 10.6 |
|  |  | 7:30 PM | 17.0 |  |  | 7:30 PM | 9.8 |
| 0 | 14 | 8:00 PM | 16.1 | 0 | 13 | 8:00 PM | 8.9 |
|  |  | 8:30 PM | 15.8 |  |  | 8:30 PM | 8.6 |
| 0 | 15 | 9:00 PM | 15.5 | 0 | 14 | 9:00 PM | 8.3 |
|  |  | 9:30 PM | 15.3 |  |  | 9:30 PM | 8.1 |
| 0 | 16 | 10:00 PM | 15.0 | 0 | 15 | 10:00 PM | 7.8 |
|  |  | 10:30 PM | 14.7 |  |  | 10:30 PM | 7.3 |
| 0 | 17 | 11:00 PM | 14.4 | 0 | 16 | 11:00 PM | 6.8 |
|  |  | 11:30 PM | 14.4 |  |  | 11:30 PM | 6.8 |
| 0 | 18 | 12:00 AM | 14.4 | 0 | 17 | 12:00 AM | 6.8 |
|  |  | 12:30 AM | 14.2 |  |  | 12:30 AM | 6.8 |
| 0 | 19 | 1:00 AM | 13.9 | 0 | 18 | 1:00 AM | 6.8 |
|  |  | 1:30 AM | 13.6 |  |  | 1:30 AM | 6.8 |
| 0 | 20 | 2:00 AM | 13.3 | 0 | 19 | 2:00 AM | 6.8 |
|  |  | 2:30 AM | 13.0 |  |  | 2:30 AM | 6.8 |
| 0 | 21 | 3:00 AM | 12.8 | 0 | 20 | 3:00 AM | 6.8 |
|  |  | 3:30 AM | 12.5 |  |  | 3:30 AM | 6.8 |
| 0 | 22 | 4:00 AM | 12.2 | 0 | 21 | 4:00 AM | 6.8 |
|  |  | 4:30 AM | 13.5 |  |  | 4:30 AM | 6.8 |
| 0 | 23 | 5:00 AM | 15.0 | 0 | 22 | 5:00 AM | 6.8 |
|  |  | 5:30 AM | 16.0 |  |  | 5:30 AM | 6.8 |
